# Adult-onset mesenteric torsion in situs ambiguus with polysplenia: the role of planned second-look laparotomy in bowel preservation

**DOI:** 10.1093/jscr/rjag964

**Published:** 2026-10-31

**Authors:** Yoshiki Komori-Valdés, Luis F Tallabs-Utrilla, Andrea Castro-Sayago, Javier Ramos-Aranda

**Affiliations:** Department of General Surgery, American British Cowdray Medical Center, Carlos Graef Fernández 154, Col. Santa Fe, Cuajimalpa, 05300, Mexico City, Mexico; Department of General Surgery, American British Cowdray Medical Center, Carlos Graef Fernández 154, Col. Santa Fe, Cuajimalpa, 05300, Mexico City, Mexico; Department of General Surgery, American British Cowdray Medical Center, Carlos Graef Fernández 154, Col. Santa Fe, Cuajimalpa, 05300, Mexico City, Mexico; Department of General Surgery, American British Cowdray Medical Center, Carlos Graef Fernández 154, Col. Santa Fe, Cuajimalpa, 05300, Mexico City, Mexico

**Keywords:** situs ambiguus, polysplenia, intestinal malrotation, mesenteric torsion, second-look laparotomy

## Abstract

Situs ambiguus, or heterotaxy, is a rare disorder of organ lateralization often accompanied by intestinal malrotation and an increased risk of midgut volvulus. Most published cases involve children with major cardiac disease, and adult presentation is exceptional. We describe a previously healthy 27-year-old woman who came to the emergency department with acute abdominal pain, peritonitis, leukocytosis, and elevated lactate. Contrast-enhanced computed tomography showed the mesenteric whirl sign, a midline liver, polysplenia, and a small splenic artery aneurysm. At laparotomy we found a 180° mesenteric torsion with vascular compromise but no necrosis, and after manual detorsion we opted for a planned second-look laparotomy at 24 h rather than resection. The second look confirmed bowel recovery, and she was discharged on day 12 without complications. A staged surgical approach can help preserve bowel in adults with aberrant mesenteric anatomy when viability is uncertain at the first operation.

## Introduction

Situs ambiguus, or heterotaxy, refers to the loss of the normal left–right asymmetry of the thoracoabdominal organs. Two phenotypes are recognized: right isomerism, which usually comes with asplenia, and left isomerism, typically associated with polysplenia; each carries its own pattern of cardiac and extracardiac anomalies [1]. Cardiac disease drives most of the morbidity in these patients, atrial isomerism, transposition of the great arteries, anomalous venous drainage, and vena caval anomalies are all part of the spectrum, and many require early surgical correction [1, 2]. Patients with asplenia are also vulnerable to infection by encapsulated bacteria and often need antibiotic prophylaxis and additional vaccination [2].

Intestinal malrotation is a third recurrent finding, carrying a real risk of obstruction or midgut volvulus [2]. Roughly 70% of patients with heterotaxy have some degree of malrotation, more commonly with right than left isomerism [3, 4]. Diagnosis is usually made in infancy, but patients with left isomerism and only minor cardiac involvement can go undiagnosed into adulthood, presenting instead with bowel obstruction, an internal hernia, or biliary disease [5]. Adult malrotation itself is uncommon, accounting for only 0.2%–0.5% of cases, and only ~15% of those present with midgut volvulus, typically around age 40 [6].

Abnormal fixation and rotation of the bowel leave the mesentery narrow-based and poorly anchored, predisposing to volvulus, and mesenteric torsion [2, 7]. Here we report an adult woman with situs ambiguus, polysplenia, and malrotation who developed acute mesenteric torsion with ischemia, successfully treated with detorsion and a planned second-look laparotomy that avoided resection.

## Case report

A previously healthy 27-year-old woman came to the emergency department with sudden, severe, diffuse abdominal pain, along with nausea and vomiting. Her only surgical history was elbow instrumentation.

On admission she was tachycardic and hypoxic, with a blood pressure of 143/94 mmHg, heart rate of 107 bpm, respiratory rate of 16/min, and oxygen saturation of 87% on room air. Laboratory work-up showed a leukocyte count of 19.2 × 10^3^/μL and a serum lactate of 8 mmol/L, both pointing toward intestinal ischemia.

Contrast-enhanced computed tomography (CT) of the abdomen and pelvis revealed compromised jejuno-ileal loops with free peritoneal fluid, situs ambiguus with polysplenia, a midline liver, and a small splenic artery aneurysm (Figs 1–3).

**Figure 1:**
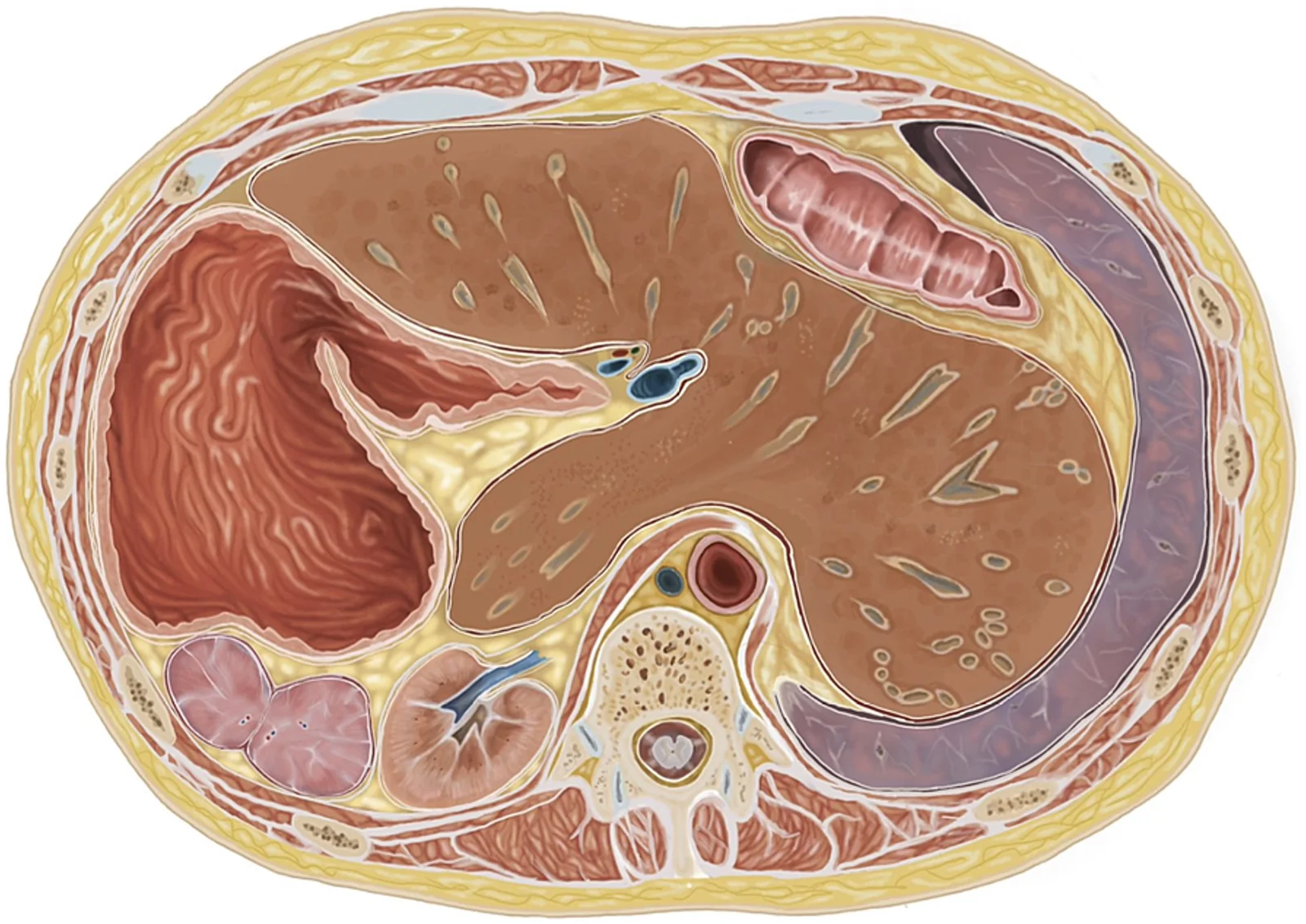
Representative illustration of the contrast-enhanced CT of the abdomen and pelvis showing an abnormal visceral arrangement consistent with situs ambiguus, with polysplenia, a medialized liver and a centrally located pancreas.

**Figure 2:**
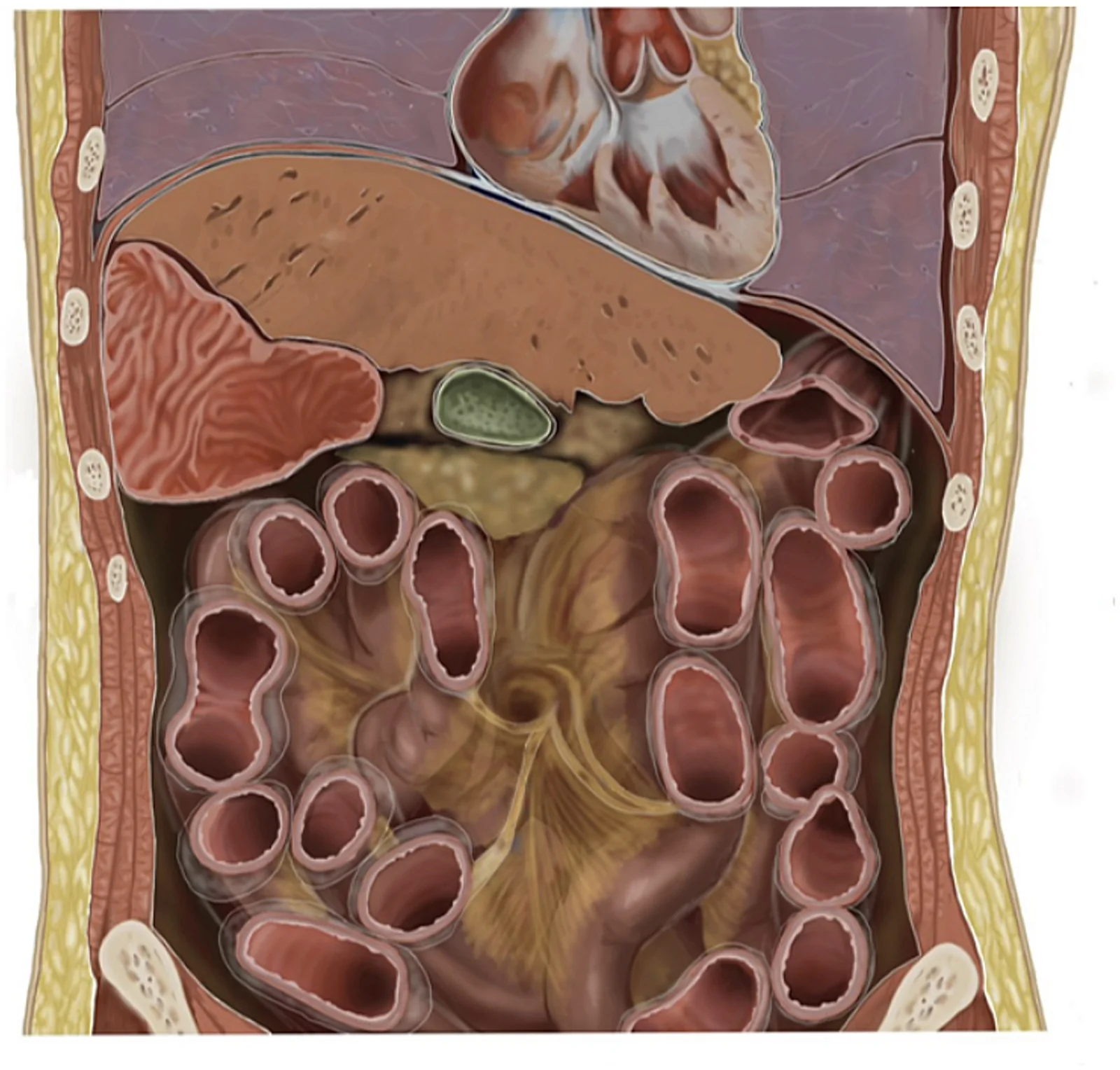
Representative illustrations of the contrast-enhanced CT of the abdomen and pelvis demonstrating intestinal malrotation with the mesenteric whirl sign, dilated bowel loops, and mural and mesenteric edema.

**Figure 3:**
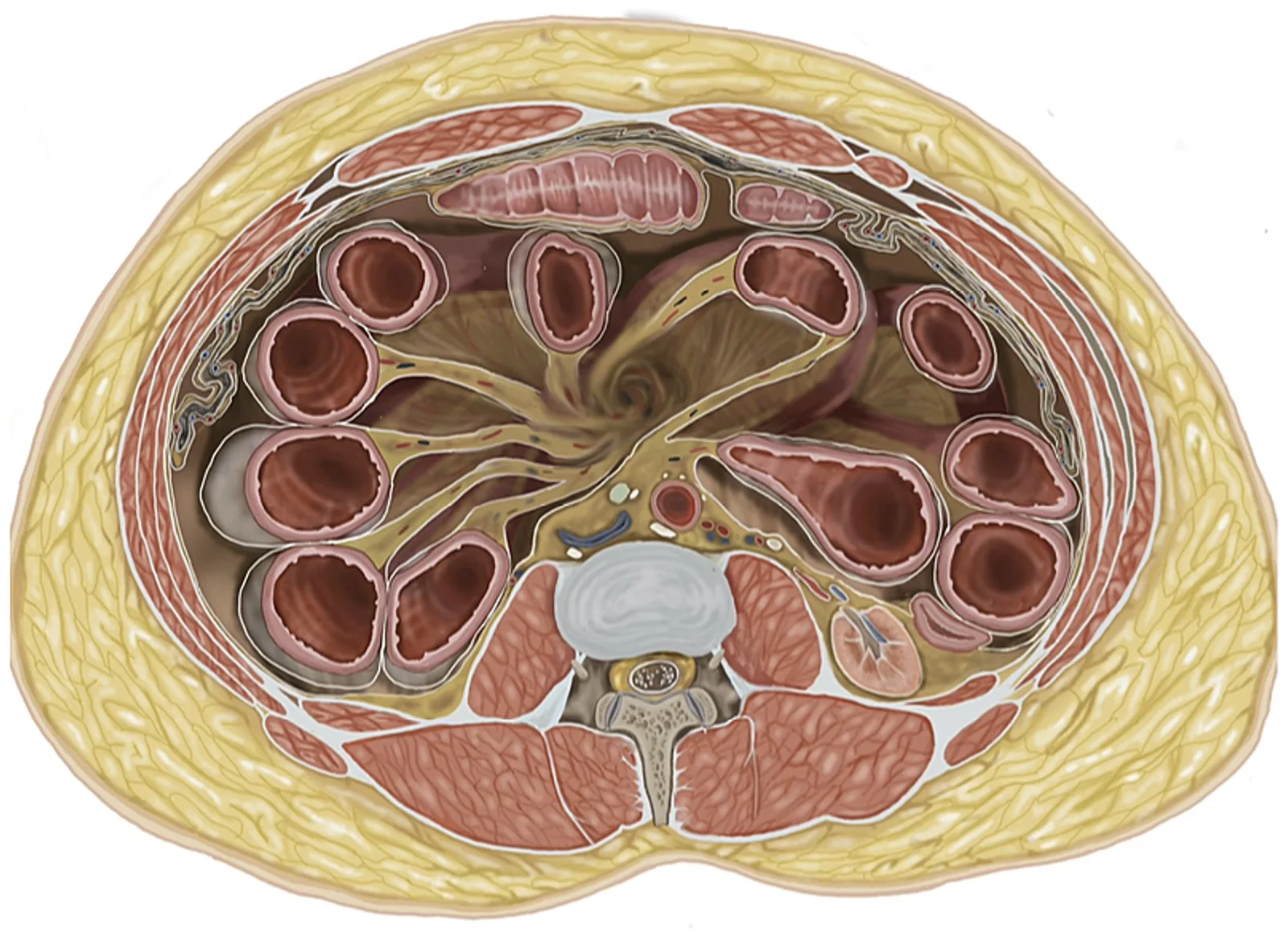
Contrast-enhanced axial CT demonstrating swirling of the mesenteric vessels around their axis (whirl sign), associated with distended, thick-walled small bowel loops and adjacent mesenteric fat stranding, findings compatible with mesenteric volvulus.

We took her for an exploratory laparotomy through a midline incision and found abundant inflammatory fluid and diffusely ischemic bowel loops. A 180° mesenteric torsion was identified, with vascular compromise but no frank necrosis. After manual detorsion, color, peristalsis, and mesenteric pulses returned. We irrigated the cavity with warm saline, placed a pelvic drain, and, since the bowel was recovering, held off on resection (Fig. 4).

**Figure 4:**
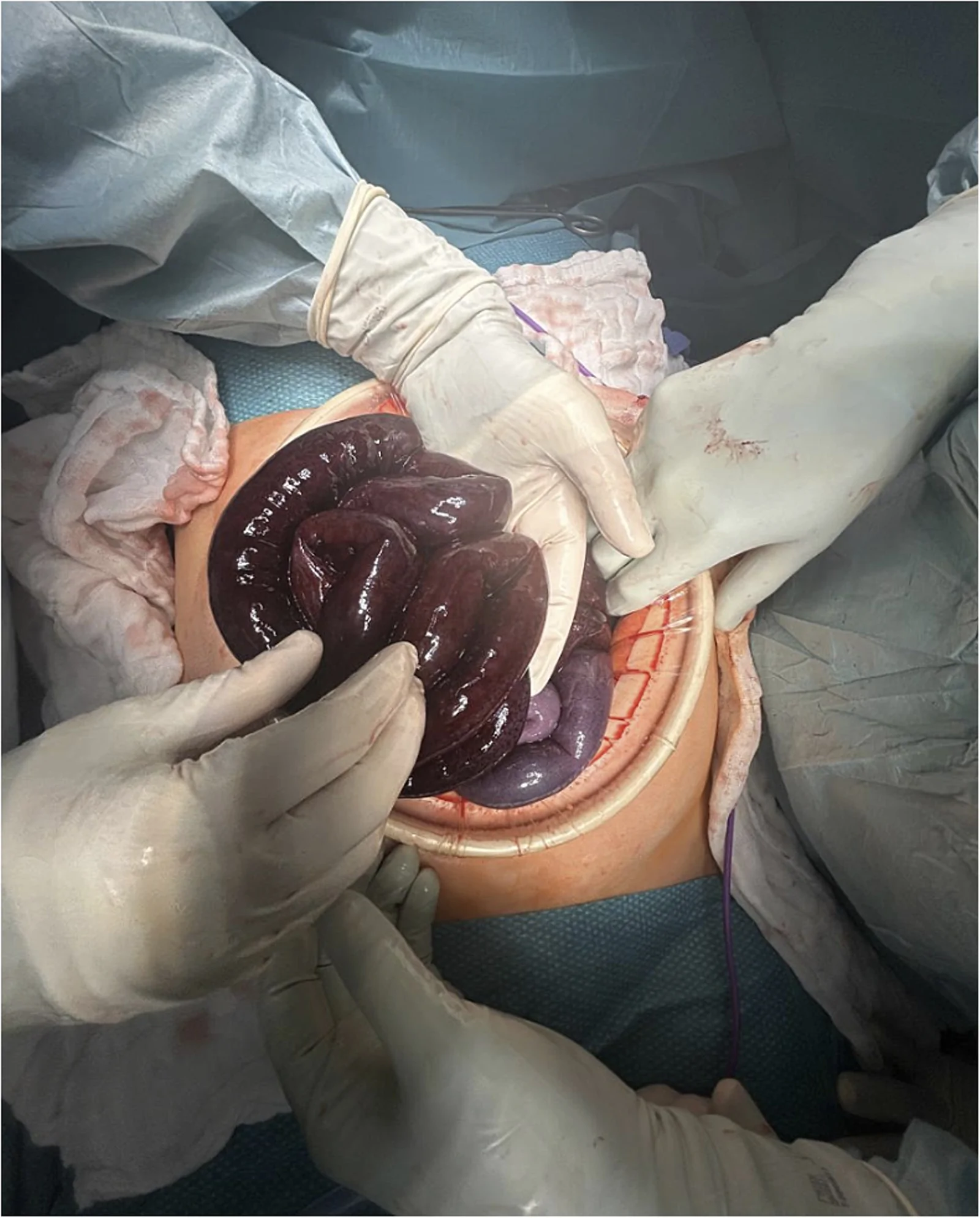
Exploratory laparotomy showing bowel loops with diffuse ischemic changes prior to detorsion.

At 24-h re-exploration the bowel was viable and the earlier ischemic changes had resolved. We closed the mesenteric defect at the ligament of Treitz and performed a prophylactic appendectomy (Fig. 5).

**Figure 5:**
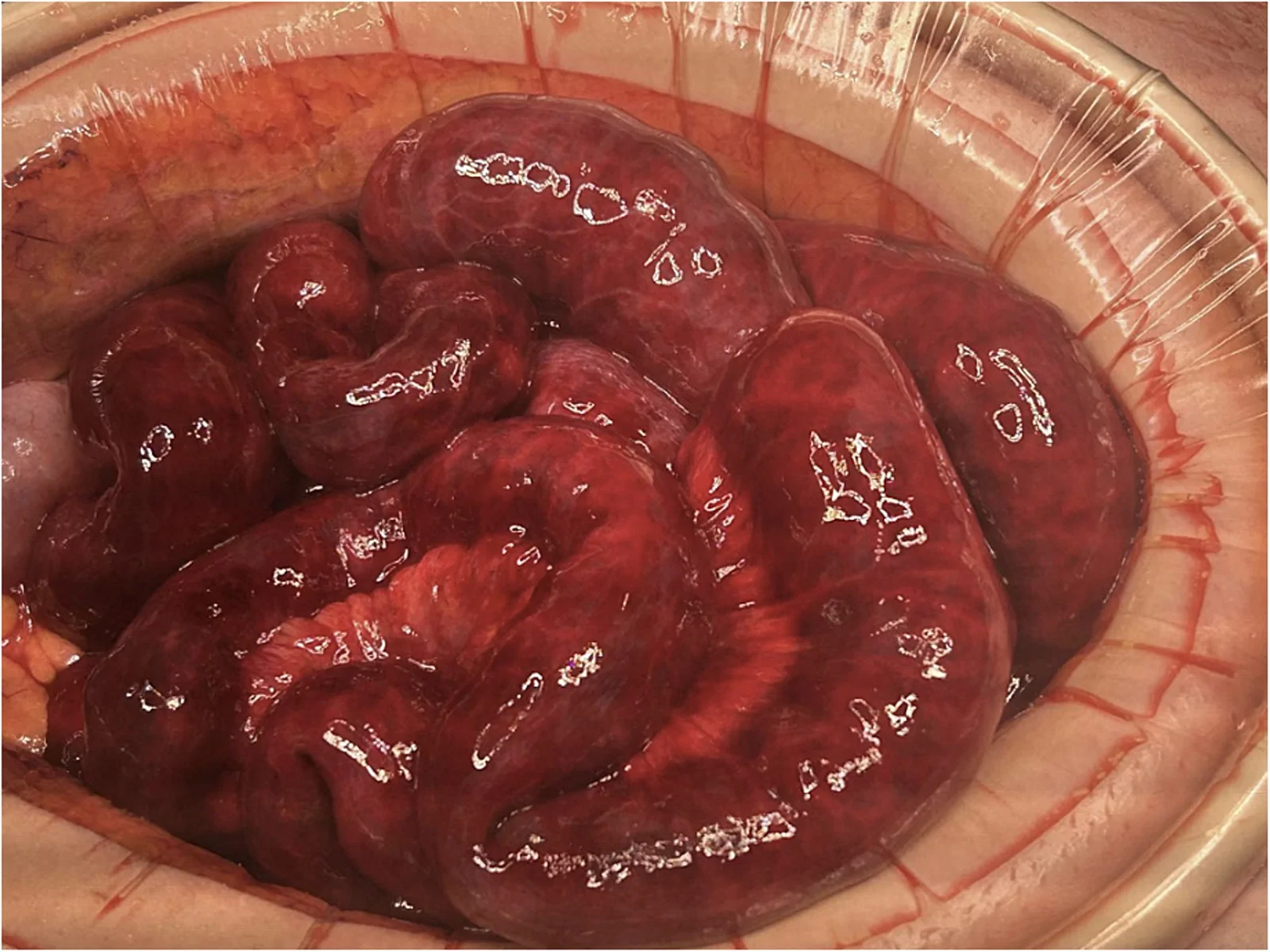
Second-look laparotomy at 24 h showing viable bowel with resolution of the initial ischemic changes.

She remained in the intensive care unit with hemodynamic support and broad-spectrum antibiotics. Lactate fell from 8 to 2.2 mmol/L within 24 h, and her leukocyte count had normalized by day 5. She was extubated on day 3, transitioned from parenteral to enteral nutrition, and discharged on day 12 in good condition, with outpatient referral to genetics and cardiology.

## Discussion

The anatomical heterogeneity of situs ambiguus complicates both diagnosis and operative planning. Much of the literature focuses on cardiac and splenic involvement; abdominal complications, just as dangerous, get less attention in adults.

Adult cases of mesenteric torsion in situs ambiguus are uncommon but not unheard of. Schwab *et al.* described an 84-year-old with malrotation and a right paraduodenal hernia who developed bowel ischemia requiring emergency surgery [8], showing how a congenital fixation defect can stay silent for decades. Our patient fits this pattern of late presentation in left isomerism: Minty *et al.* reported a 39-year-old woman with polysplenia and midgut volvulus and no significant cardiac anomalies, likely why she reached adulthood undiagnosed [5]. The largest review, by Butterworth and Butterworth, pooled 92 adult cases of malrotation with midgut volvulus: CT made the diagnosis in 67%, resection was needed in nearly one in five patients (mean 121 cm resected), and mortality reached 5% [6]. Our patient avoided resection, likely thanks to early imaging, prompt exploration, and a second look that confirmed viability first.

A few radiological clues should raise suspicion: an inverted relationship between the superior mesenteric artery and vein, a non-retroperitoneal duodenum, a displaced duodenojejunal junction, multiple or absent spleens, a midline liver, and, in acute volvulus, the mesenteric whirl sign [7, 9]. Our patient had all of them, and recognizing the pattern matters: unusual anatomy can otherwise mislead the radiologist and delay surgery.

Planned second-look laparotomy is what made the difference here. Meng *et al.* showed years ago that this approach spares unnecessary resections by letting the surgeon assess recovery before committing to surgery [10], and the updated World Society of Emergency Surgery guidelines recommend re-exploration at 24–48 h when viability is doubtful, sooner if the patient's condition worsens [11]. We settled on 24 h as a compromise: enough time for microcirculatory recovery, but not so long that a deteriorating segment could be missed.

At the second look we also closed the mesenteric defect and performed a prophylactic appendectomy, reasoning that future right-lower-quadrant pain would be a diagnostic nightmare in a patient with atypical anatomy. We deliberately stopped short of a formal Ladd's procedure: she was hemodynamically stable, there were no obstructive duodenal bands, and the evidence on prophylactic Ladd's in heterotaxy is mixed, with complications driven mainly by associated cardiac disease and functional asplenia [12, 13]. This decision should be individualized. When possible, studying the CT angiogram closely beforehand, and adapting the dissection accordingly, is the most practical lesson here [2].

Situs ambiguus is rare in adults, but it belongs on the differential for anyone with an acute abdomen and CT signs of malrotation. A planned second-look at 24 h, paired with closure of the mesenteric defect and a prophylactic appendectomy, gives the bowel a chance to recover and the surgeon a chance to reassess before committing to resection.
